# Low mutation and neoantigen burden and fewer effector tumor infiltrating lymphocytes correlate with breast cancer metastasization to lymph nodes

**DOI:** 10.1038/s41598-018-36319-x

**Published:** 2019-01-22

**Authors:** Zhigang Wang, Wei Liu, Chong Chen, Xiaolin Yang, Yunping Luo, Bailin Zhang

**Affiliations:** 1 Department of Biomedical Engineering, Institute of Basic Medical Sciences and School of Basic Medicine, Peking Union Medical College and Chinese Academy of Medical Sciences, Beijing, China; 2 Department of Anatomy and Histology, Institute of Basic Medical Sciences and School of Basic Medicine, Peking Union Medical College and Chinese Academy of Medical Sciences, Beijing, China; 3 Department of Immunology, Institute of Basic Medical Sciences and School of Basic Medicine, Peking Union Medical College and Chinese Academy of Medical Sciences, Beijing, China; 4 Department of Breast Surgery, National Cancer Center/National Clinical Research Center for Cancer/Cancer Hospital, Chinese Academy of Medical Sciences and Peking Union Medical College, Beijing, China

## Abstract

Lymph node metastasis is of major prognostic significance for breast cancer. Lymph node metastasis arises at a very early stage in some patients. Using the data downloaded from the TCGA database, we studied the differences between primary tumors with and without lymph node metastasis at the multi-omics level using bioinformatics approaches. Our study found that low mutation and neoantigen burdens correlated with lymph node metastazation of breast cancer. All three conserved domains in TP53 were mutated in lymph node-negative breast cancers, whereas only one domain was mutated in lymph node-positive samples. Mutations in microtubule-related proteins appear to help immune cells recognize tumors and inhibit their lymph node metastasis. Destroying microtubule-related proteins is a potential therapeutic strategy to inhibit lymph node metastasis of breast cancer. As the neoantigens specifically present in lymph node-positive breast cancers, MAPK10, BC9L, TRIM65, CD93, KITLG, CNPPD1, CPED1, CCDC146, TMEM185A, INO80D, and PSMD11 are potential targets for vaccine design. In the tumor microenvironment, reduced numbers of effector immune cells, especially activated memory CD4+ T cells and activated mast cells, facilitate breast cancer metastasis to the lymph nodes. According to transcriptome data, lymph node metastasis was mostly driven by gene mutation rather than by gene expression. Although differential gene expression analysis was based on lymph node metastasis status, many genes were shown to be differentially expressed based on estrogen receptor status.

## Introduction

Breast cancer is the most frequently occurring cancer in women and has become a major public health problem. The worldwide incidence of female breast cancer has been predicted to reach approximately 3.2 million new cases per year by 2050^1^.

Lymph node metastasis is of major prognostic significance for breast cancer^2^. The presence and number of lymph node metastases are associated with compromised survival in patients with other types of cancer, such as papillary thyroid cancer^3^. Metastasis is caused by complex interactions that involve many factors, including molecular factors triggered by tumor cell proliferation, cytokine production and expansion, tumor microenvironmental changes, and other mechanical factors inside the tumor and their interactions with host tissues^4^.

The transitional view indicates that tumor metastasis is the result of an accumulation of mutations, especially mutations in metastasis genes. A study by Simpson *et al*. showed that the tumor mutation burden increases the presentation of neoantigens that stimulate immune tumor recognition, resulting in improved immunotherapy outcomes in melanoma and other cancers^5^. A higher mutation burden and mutant allele fraction of circulating tumor DNA corresponds to a worse progression-free survival in metastatic breast cancer patients^6^. Mansfield *et al*. observed a higher mutation burden in metastatic lesions^7^. However, the relationship between mutation and neoantigen burden of primary breast cancer and lymph node metastasis is not known.

Tumor-infiltrating lymphocytes (TILs) are associated with the response to neoadjuvant chemotherapy in triple-negative breast cancer (TNBC) and HER2-positive breast cancer^8^. Pan-cancer immunogenomic analyses have revealed that many TILs related to adaptive immunity are associated with a good prognosis, including activated CD8+ T cells, effector memory T cells and central memory CD8+ T cells, and effector memory CD4+ T cells, whereas MDSCs and Tregs are associated with a poor prognosis^9^. Therefore, studying TILs that are highly enriched in non-lymph node metastasis breast cancers can provide clues for slowing tumor progression.

In clinical practice, we noted that breast cancer is highly heterogeneous in its pathological characteristics. Some patients have no lymph node metastasis, even when the primary tumors are relatively large, while others have lymph node metastasis at a very early stage. To investigate the mechanism of lymph node metastasis in breast cancer, we downloaded whole exome sequencing data and RNA-seq data from 243 samples from the TCGA project and assessed the tumor itself and tumor microenvironmental characteristics, such as the mutation burden, neoantigens, tumor heterogeneity, TILs and gene expression. Interestingly, we noted that a high mutation burden and neoantigen burden can suppress lymph node metastasis of breast cancer. Most of the lymph node-negative specific mutations are in proteins associated with microtubules. In other words, destroying microtubule-related protein structures may help inhibit lymph node metastasis in breast cancer. For TP53, the distribution of mutation hotspots in the lymph node-positive group was clearly distinct from that in the lymph node-negative group. We analyzed the neoantigen origin proteins specifically present in the lymph node metastasis group, which suggested potential target therapies for inhibiting breast cancer metastasis. As expected, the fraction of effector TILs is higher in samples with no lymph node metastasis than in samples with lymph node metastasis. In particular, the proportions of activated memory CD4+ T cells and activated mast cells in the lymph node-negative group were both double those in the lymph node-positive group.

## Results

### Sample demographic statistics

The publicly available 1098 BRCA clinical information in the TCGA database was used as the primary source. Using the criteria in the methods section, there were 128 LN-negative samples and 115 LN-positive samples. The demographic characteristics are shown in Table 1.

**Table 1:** Demographic characteristics of TCGA samples.

	LN-negative	LN-postive	p
N	128	115	
Race (%)			0.355
NA	3 (2.3)	0 (0.0)	
American Indian or Alaska native	0 (0.0)	1 (0.9)	
Asian	6 (4.7)	5 (4.3)	
Black or African American	23 (18.0)	17 (14.8)	
White	96 (75.0)	92 (80.0)	
Number of positive lymphnodes by HE (mean (sd))	0.00 (0.00)	7.13 (5.71)	<0.001
Progesterone receptor status (%)			0.035
NA	9 (7.0)	10 (8.7)	
Indeterminate	0 (0.0)	1 (0.9)	
Negative	54 (42.2)	29 (25.2)	
Positive	65 (50.8)	75 (65.2)	
Estrogen receptor status (%)			0.01
NA	8 (6.2)	10 (8.7)	
Indeterminate	0 (0.0)	1 (0.9)	
Negative	46 (35.9)	20 (17.4)	
Positive	74 (57.8)	84 (73.0)	
HER2 immunohistochemistry receptor status (%)			0.026
NA	15 (11.7)	26 (22.6)	
Equivocal	28 (21.9)	12 (10.4)	
Indeterminate	0 (0.0)	1 (0.9)	
Negative	69 (53.9)	57 (49.6)	
Positive	16 (12.5)	19 (16.5)	
Therapy types (%)			0.019
NA	36 (28.1)	24 (20.9)	
Ancillary	1 (0.8)	1 (0.9)	
Chemotherapy	55 (43.0)	73 (63.5)	
Chemotherapy and hormone therapy	0 (0.0)	1 (0.9)	
Hormone therapy	34 (26.6)	12 (10.4)	
Immunotherapy	1 (0.8)	1 (0.9)	
Targeted molecular therapy	0 (0.0)	1 (0.9)	
Other	1 (0.8)	2 (1.7)	
Pathologic stage (%)			<0.001
Stage II	3 (2.4)	1 (0.9)	
Stage IIA	106 (82.8)	8 (7.0)	
Stage IIB	15 (11.7)	26 (22.6)	
Stage III	0 (0.0)	2 (1.7)	
Stage IIIA	0 (0.0)	53 (46.1)	
Stage IIIB	4 (3.1)	3 (2.6)	
Stage IIIC	0 (0.0)	22 (19.1)	
Age at initial pathologic diagnosis (mean (sd))	54.87 (9.19)	52.43 (9.08)	0.039
Vital status follow up (%)			0.142
NA	2 (1.7)	3 (2.8)	
Alive	111 (91.7)	89 (83.2)	
Dead	8 (6.6)	15 (14.0)	
OS time (mean (sd))	1108.70 (1069.91)	1416.06 (1426.87)	0.091

### Mutation burden in relation to lymph node metastasis

**Figure 1:**
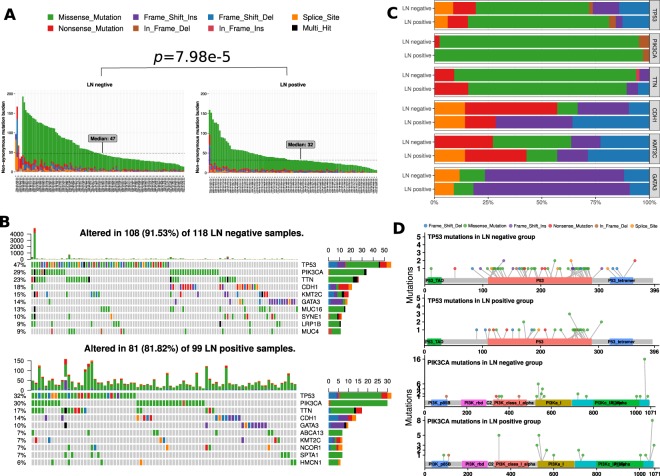
Landscape of Mutations in Breast Cancer. (**A**) Per-sample non-synonymous mutation burden for (left) LN-negative and (right) LN-positive groups. Mutation types, including missense, frame-shift ins/del, in-frame ins/del, nonsense (stop-gain) and splice-site, are colored according to the legend. The graph shows that mutation burdens are significantly different between LN-negative and LN-positive groups by the Wilcox rank sum test (*p* < 0.05). (**B**) Color-coded matrix of individual mutations of the top 10 most mutated genes for (top) LN-negative and (bottom) LN-positive groups. In cases in which multiple mutations per gene were found in a sample, it is colored black. The right stacked bar plot of each figure displays the number of variant types. (**C**) Stacked bar plot shows the fraction of variant types in LN-negative and LN-positive groups. (**D**) Mutation spots on the TP53 and PIK3CA proteins. The number of mutations in each spot is shown on the y-axis. Mutation types are colored according to the legend. P53 mutation hotspots are dispersed and (top) in the P53, TAD and tetramer domains in the LN-negative group, but (bottom) only in the p53 domain in the LN-positive group. The PIK3CA mutation hotspots are clustered in specific areas.

In general, a malignant tumor, such as a tumor with lymph node metastasis, was considered to have a high mutation burden. We first asked whether the non-synonymous mutation burden could distinguish LN-negative and LN-positive groups. The somatic mutations detected by the mutect2 software of 118 LN-negative samples and 99 LN-positive samples were available. Interestingly, as shown in Fig. 1A, the non-synonymous mutation burden of the LN-negative group (median 47) was significantly higher (Wilcox rank-sum test *p* < 0.0001) than that of the LN-positive group (median 32). As high TMB may be associated TNBC, we stratified the data into 2 groups, TNBC group and non-TNBC group. TMBs were compared between LN-negative and LN-positive groups in each stratification. For the TNBC stratification, the Wilcox rank sum test was used and a p-value of 0.008 was detected. For non-TNBC samples, the p value was found to be 0.012.

### Highly mutated genes with distinct mutation patterns

Mutational patterns of highly mutated genes were distinct between the LN-negative and LN-positive groups. In the top 10 mutated genes of the LN-negative and LN-positive groups, TP53, PIK3CA, TTN, CDH1, GATA3, and KMT2C are shared (Fig. 1B). More nonsense (stop-gain) and fewer frame-shift-deletion mutations on the CDH1 gene were in the LN-negative group than in the LN-positive group. We also noted one nonsense mutation in PIK3CA in the LN-negative group (Fig. 1C). As TP53 is a tumor suppressor gene, the mutation spots were discrete. PIK3CA is a proto-oncogene, and the mutation spots were clustered (Fig. 1D). We noted that all three conserved domains on TP53 were mutated in the LN-negative group. However, only one conserved domain was mutated in the LN-positive group. The mutated spot distributions on PIK3CA were similar between the LN-negative and LN-positive groups (Fig. 1D).

### Almost all genes with significantly differential mutation rates are specific to the LN-negative group

We selected genes with a significantly different mutation rate between LN-negative and LN-positive groups, as shown in Table 2. The numbers in the second and third columns are number of samples with mutations for each gene. All of the genes, except PLD5, were highly mutated in the LN-negative group.

**Table 2:** Genes with significantly different mutation rate between LN-negative and LN-positive groups.

Gene	LN-positive (n = 99)	LN-negative (n = 118)	p-value
DST	0	10	0.002187
PCNT	0	9	0.004294
LRP1B	1	11	0.00713
HERC2	0	8	0.008435
RB1	0	8	0.008435
ZDBF2	0	8	0.008435
PCDH15	0	7	0.01658
PREX2	0	7	0.01658
TNIK	0	7	0.01658
XIRP2	0	7	0.01658
ZNF536	0	7	0.01658
DNAH7	1	9	0.02326
VPS13C	1	9	0.02326
TP53	32	56	0.02681
MUC16	4	15	0.02957
BIRC6	0	6	0.03265
BRCA1	0	6	0.03265
CECR2	0	6	0.03265
CNTNAP5	0	6	0.03265
DUSP27	0	6	0.03265
PCDH19	0	6	0.03265
PDE4B	0	6	0.03265
RP1	0	6	0.03265
SI	0	6	0.03265
TPR	0	6	0.03265
TRPS1	0	6	0.03265
PLD5	4	0	0.04189

**Figure 2:**
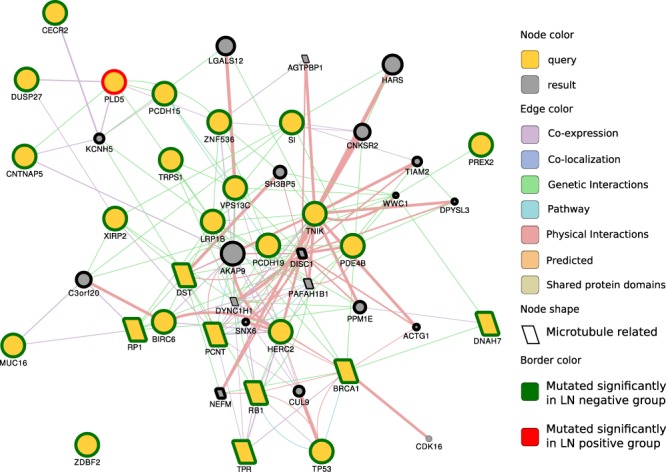
GeneMANIA network of genes with significantly different mutation rates in LN-negative and LN-positive groups. The query genes are yellow, and the resulting genes are gray. A larger node size indicates that the gene is more likely to be functionally related to query genes in the network. Gene interaction types, including physical interaction, genetic interaction and co-expression, are colored according to the legend. The green border indicates that the gene is highly mutated in the LN-negative group. The red border indicates that the gene is highly mutated in the LN-positive group. A diamond shape indicates that the nodes are microtubule-related genes. The graph shows that most genes with significantly different rates, except PLD5, are highly mutated in the LN-negative group.

To investigate the functional association of the genes with a significantly different mutation rate, we analyzed them with the GeneMANIA plugin in the Cytoscape software (Fig. 2). The yellow genes are query genes, while the gray genes are related to the query genes. Most of the network interactions were physical interactions, genetic interactions, or co-expression. The largest functional group genes in the network was related to microtubules (shown with a diamond shape in Fig. 2), such as microtubule cytoskeleton organization, microtubule-associated complex, and microtubule binding. The involved genes included BRCA1, PCNT, BIRC6, RP1, RB1, TRP, DNAH7, PAFAH1B1, DYNC1H1, DISC1, and AGTPBP1.

### Neoantigen burden is low in LN-positive samples, but neoantigen origin proteins may be potential vaccine targets

**Figure 3:**
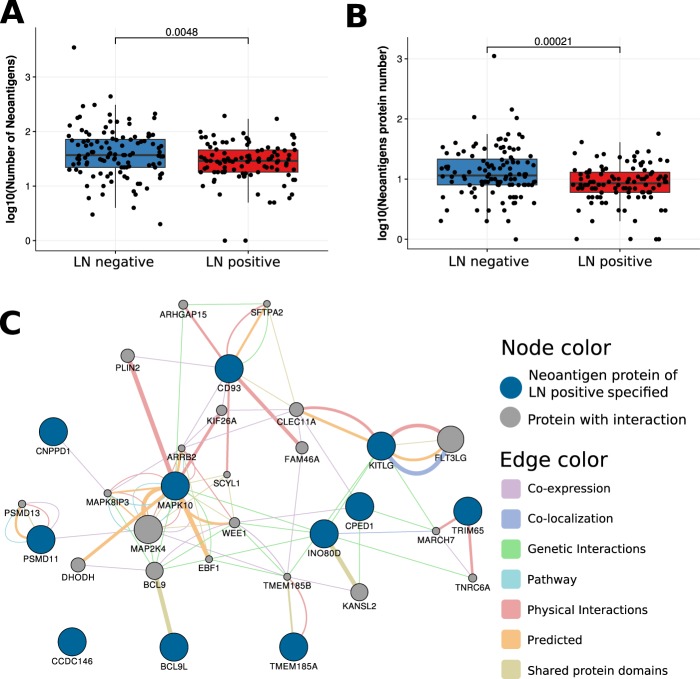
Neoantigen comparison between LN-negative and LN-positive breast cancer. (**A**) The boxplot of the number of neoantigen peptides. (**B**) The boxplot shows the number of neoantigen-related proteins. The graph shows that neoantigen burdens are significantly different between LN-negative and LN-positive groups by the Wilcox rank sum test (*p* < 0.005). (**C**) Network of neoantigen origin proteins specified in LN-positive breast cancer samples. LN-positive specific neoantigen origin proteins include the MAPK family, the BCL family, TRIM65, and CD98.

The LN-positive samples had significantly lower (Wilcox rank sum test *p* < 0.005) neoantigen burden (Fig. 3A) and neoantigen origin protein burden (Fig. 3B) than samples from the LN-negative group. The neoantigen origin proteins (N = 11) that occurred in only the LN-positive group were closely connected (Fig. 3C). LN-positive-specific neoantigen proteins included MAPK10, BC9L, TRIM65, CD93, KITLG, CNPPD1, CPED1, CCDC146, TMEM185A, INO80D, and PSMD11. MAPK is a type of protein kinase that is involved in directing cellular responses to a diverse array of stimuli, such as mitogens, osmotic stress, heat shock and proinflammatory cytokines^10^. MAPKs regulate cell functions, including proliferation, gene expression, differentiation, mitosis, cell survival, and apoptosis. BCL9L (B-cell CLL/lymphoma 9 like) protein shares a conserved domain with BCL9, which is related to intestinal tumor progression. TRIM65 can trigger -catenin signaling via ubiquitylation of Axin1 to promote hepatocellular carcinoma^11^.

### LN-negative samples have high heterogeneity

**Figure 4:**
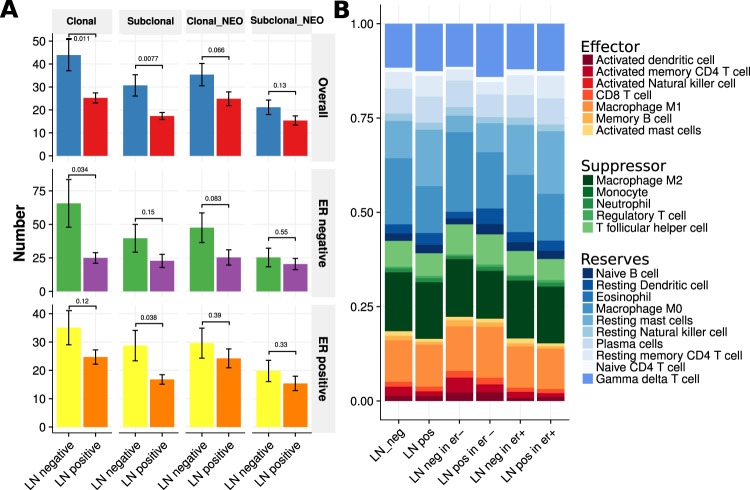
Tumor heterogeneity and infiltrated cells. (**A**) Clonal and subclonal number and neoantigen origin comparison between LN-negative and LN-positive group (top) overall samples. (middle) Results for ER-negative samples. (bottom) Results for ER-positive samples (two-sided Wilcox rank sum test). The height of the bar is the clonal number mean, and the error bar is the standard error. (**B**) Immune infiltrate cell fractions.

Tumor heterogeneity and clonality of mutations within lesions are deemed responsible for relapses in malignancies and present challenges for targeted therapy. Therefore, we compared clonality and neoantigen origin clonal information between the LN-negative and LN-positive groups (Fig. 4A). Obviously, in the overall samples, ER-negative samples, or ER-positive samples, there were more clonal and subclonal samples in the LN-negative group. We also noted that the number of neoantigens from clonal and subclonal samples had the same trend, although many were not statistically significant. The tumor composition of the ER-negative group was more complex than that of the ER-positive group (Fig. 4A middle vs bottom). This result could help to explain why the ER-negative samples were more malignant.

### Activated memory CD4+ T cells and mast cells heavily infiltrated samples from the LN-negative group

TILs include T cells, B cells, natural killer cells, macrophages, neutrophils, dendritic cells, mast cells, eosinophils, and basophils. Tumor-infiltrating immune cells can often be found in the stroma and within the tumor itself. Their functions can dynamically change throughout tumor progression and in response to anticancer therapy. TILs are implicated in killing tumor cells. The presence of lymphocytes in tumors is often associated with a better clinical outcome.

We classified 22 immune cell types into three groups: effectors, suppressors, and reserves (Table S1 and Fig. 4B). In overall samples, ER-negative samples, and ER-positive samples, there were more effector immune cells and fewer reserve cells in the LN-negative group. In particular, the activated memory CD4+ T cell fraction in the LN-negative group was approximately 2.4%, which was double that of the LN-positive group. The fraction of activated mast cells was higher in the LN-negative group. The number of CD8+ T cells and activated dendritic cells in the LN-negative group was slightly higher than that of the LN-positive group. There were no obviously different suppressor cell fractions between the LN-negative group and the LN-positive group. Resting mast cell fraction was higher in the LN-positive group than in the LN-negative group.

### Lymph node metastasis of breast cancer is likely driven by mutations but not by changes in gene expression

**Figure 5:**
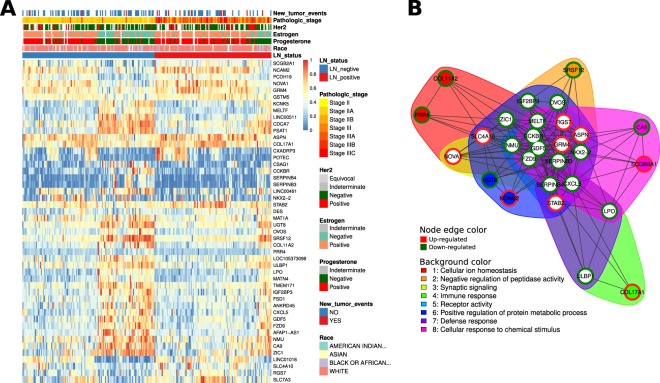
Gene expression spectrum. (**A**) Heatmap of differentially expressed genes between LN-negative and LN-positive BRCA samples. Expression values are represented by a 0–1 scale. Clinical characteristics are colored according to the legend. (**B**) Functional clustering of differentially expressed genes. The red node color indicates upregulated gene expression in the LN-positive group, whereas the green node color indicates the opposite.

DESeq2, TCGAanalyze_DEA and limma methods were used to select 598, 456 and 866 genes as differentially expressed genes (DEGs), respectively. Forty-eight DEGs were shared by three methods. Although stringent criteria were used to select DEGs, the heatmap showed that some genes were expressed unstably in a group. We noted that the instability of expression could mostly be explained by estrogen status. The estrogen status was often the same as the progesterone and estrogen status but not the HER2 status (Fig. 5A).

Functional clustering shows that the DEG functions are associated with ‘immune response’, ‘defense response’ and ‘cellular response to chemical stimulus’, among others (Fig. 5B).

## Discussion

To study the mechanism of lymph node metastasis in breast cancer, we analyzed exome sequencing and RNA sequencing data from more than 200 samples from the TCGA project. Our results revealed a number of associations for breast cancer lymph node metastasis, such as non-synonymous mutation burden, neoantigen burden, significantly different gene mutation rates, mutation hotspot distribution on TP53, tumor heterogeneity, neoantigen origin proteins and differentially expressed genes.

First, we observed that breast cancer samples with lower mutation and neoantigen burden are more likely to have lymph node metastasis. The cumulative theory of mutations suggests that tumors are caused by increased mutations. With the increase in the mutation load, the original normal cells gain the ability to indefinitely differentiate and form tumor cells. The higher the mutation load, the higher the malignancy of the tumor. The trend in tumor metastasis is an important indicator for evaluating the malignancy of the tumor. Cazier *et al*. found that in bladder cancer patients, the mutation load was correlated with clinical pathology. A high mutation load can help identify lesions with a high risk of invasiveness in early or poorly differentiated tumors^12^. However, we found that breast cancer samples with no lymph node metastasis have a higher mutation burden. These tumor cells can be regarded as foreign substances; therefore, non-specific immune cells are more likely to target cells with a large number of mutations. In our analysis, it was found that in the non-lymph node metastasis group, the level of activated dendritic cells was higher than that of lymph node metastases, and these cells could stimulate innate immunity. The innate immune system cleared the highly mutated cells quickly, and they could not metastasize. Our results were consistent with the results of Birkbak *et al*. They found that, in TCGA ovarian cancer samples, a small number of non-synonymous mutations suggested that patients have chemotherapy resistance and a shorter progression-free survival and overall survival, while a large number of homozygous mutations predicted a better prognosis for ovarian cancer patients^13^.

Many genes with a significantly different mutation rate between LN-positive and LN-negative groups were related to microtubules. Tubulin and microtubule-associated proteins may play a role in a series of cellular stress responses, thereby helping cancer cell survival^14^. The tubulin family is the target of tubulin-based chemotherapeutic drugs, which inhibit the dynamics of the mitotic spindle causing mitotic arrest and cell death. Changes in microtubule stability and the expression of different tubulin isoforms as well as altered post-translational modifications have been reported to be involved in a variety of cancers^15^.

Somatic mutation-induced tumor-specific antigens (neoantigens) have become key targets of immunotherapy. Neoantigen burden can be a biomarker in cancer immunotherapy and provide an incentive for the development of novel therapeutic approaches that selectively enhance T cell reactivity against this class of antigens^16^. We found that the neoantigen peptide burden was significantly higher in the non-lymph node metastasis group than in the lymph node metastasis group. More than one neoantigen peptide can come from one protein. When comparing neoantigen origin protein burden, the difference is more pronounced. However, we noted that the neoantigen origin clonal fraction in the 2 groups was almost the same. Therefore, if a tumor vaccine was used against the neoantigen in the clone, there would be no difference in the response rate between the two types of breast cancer.

A neoantigen-targeting vaccine showed promise in several preclinical and clinical studies. However, to date, neoantigen vaccine studies have involved only tumors with a high mutation burden. In reality, T cells that specifically target neoantigens do not always recognize tumor cells. In other words, corresponding mutations do not produce MHC-presenting epitopes^17^. In our study, we filtered neoantigen-associated proteins in lymph node metastasis samples. It can be hypothesized that drugs targeting these proteins can inhibit lymph node metastasis in breast cancer. These proteins include MAPK10, BCL9L, TRIM65, CD93, KITLG, CNPPD1, CPED1, CCDC146, TMEM185A, INO80D, and PSMD11. Using mass spectrometry technology, Maurizio and his colleagues also found that CD93 is an antigen bound by 4E1 and mapped the recognized epitope. CD93 is a transmembrane protein that is heavily glycosylated and preferentially expressed in the vascular endothelium. CD93 silencing impairs human endothelial cell proliferation, migration, and sprouting. They revealed that 4E1 was a novel antiangiogenic antibody and identified CD93 as a new target suitable for antiangiogenic therapy^18^. This study suggested that the proteins we listed give us clues for potential immunotherapy targets. The neoantigen of CD93 only occurs in breast cancer with lymph node metastasis and indicates a close relationship between angiogenesis and lymph node metastasis.

It can be understood that the tumor suppressor gene TP53 has a discrete distribution of mutations and that mutations in the proto-oncogene PIK3CA cluster into hotspots. These two genes are highly mutated in both the lymph node metastasis group and the nonmetastatic group. The study by Kotoula *et al*. showed that TP53 and PIK3CA mutations appear to have diverse effects on the outcome of early breast cancer patients, according to whether or not these genes were comutated^19^. We found that 14 (13.0%) samples in the nonmetastasis group were comutated in these 2 genes. Correspondingly, the number of comutated samples in the lymph node metastasis group was 9 (9.1%). The proportion was low. Another finding is that in breast cancer samples with lymph node metastasis, the mutation hotspots in TP53 are only discretely distributed in the p53 (DNA-binding) domain, which is consistent with the previous study that most cancer somatic mutations are located in the DNA-binding domain^20^. In the non-lymph node metastasis group, mutations are widely distributed on three conserved domains, P52_TAD (natively unfolded amino-terminal transactivation domain), P53 DNA-binding and P53_tetramer (tetramerization). This phenomenon may be a biomarker for good prognosis.

The TIL status has been recently proposed to predict the clinical outcome of patients with breast cancer. TILs are independent positive prognostic indicators of survival time for neoadjuvant anti-HER2 therapy and chemotherapy for early breast cancer patients^21^. In the future, TILs should be considered a prognostic marker of clinical therapies for HER2-positive BC^22^. We found that the activated memory CD4+ T cell fraction in the LN-negative group was approximately 2.4%, which is double that of the LN-positive group. Lucas *et al*. compared primary and metastatic thyroid cancer and noted that LN metastasis is enriched with activated immune cells^23^. Unlike their study, we compared primary cancer, with one group having lymph node metastasis in the early stage and the other group having no lymph node metastasis, even though the primary tumor was relatively large. Our results suggested that metastasis ability was not gained by tumor growth and differentiation, and tumors with innately metastasis ability use a different intrinsic mechanism.

The weakness of this study is that we only analyzed a limited sample size per group. However, tumors are highly heterogeneous diseases. Our data suggested that there are trends that may not be observed in some other specific samples. In addition, many conclusions are not necessarily suitable for other types of tumors.

## Methods

The publicly available BRCA datasets were downloaded from the TCGA project^24^ using the TCGAbiolinks package^25^ from Bioconductor. We selected 30- to 70-year-old females with no positive lymph nodes by HE, stage II, IIA, IIB, or IIIB, TNM categories^26^ of T4N0M0, T4bN0M0, T3N0M0, T3N0(i-)M0, T2N0M0 or T2N0(i-)M0 samples as the LN-negative group. Using these criteria, the samples in the LN-negative group with very early stage or too small of a tumor size were excluded. Samples from 30- to 70-year-old females with >3 positive lymph nodes, stage IIIA or IIIC, TNM categories of T1cN1M0, T1cN1MX, T2bN1M0, T2N1aM0, T2N1bM0, T2N1M0, T1bN3aM0, T1cN1aM0, T2N1M0, T1bN3aMx, T1cN2aM0, T1cN2aMx, T1cN2M0, T1cN3aMx, T1N2M0, T2N2aM0, T2N2aMx, T2N2M0, T2N3aM0, T2N3aMx, T2N3bM0, T2N3bMx, T2N3cM0, T2N3M0, T2N3Mx, T4bN1bM0, T4bN1M0, T3N2M0 and T3N3M0 were classified as the LN-positive group. Samples with very a late stage or too large of a tumor size were excluded.

The somatic mutations detected by the mutect2^27^ software were downloaded from the TCGA project. The mutation dataset for 118 LN-negative samples and 99 LN-positive samples were available. The synonymous variants and variants in the intergenic or noncoding regions were filtered out for mutation burden analysis. The maftools^28^ package was used for mutation spectrum visualization. A chi-square test from the R chi sq. test function^29^ was used to compare the sample mutation rates between the LN-negative and LN-positive groups.

To investigate the functional association of the genes with significantly different mutation rates, we utilized the GeneMANIA plugin^30^ in the Cytoscape^31^ software (version 3.6.0; National Institute of General Medical Sciences, Seattle, WA, USA) based on a large set of functional association data, including protein and genetic interactions, co-expression, co-localization pathways, and protein domain similarity.

The neoantigens for each sample, clonality and neoantigen origin clonal information were downloaded from TCIA 9 (The Cancer Immunome Atlas, https://tcia.at/) project. In the TCIA pipeline, HLA alleles were determined from RNA-sequencing data using Optitype^32^. Mutated protein peptides of 8–11 amino acids in length were analyzed with NetMHCpan^33^ to estimate their binding affinity to the HLA alleles. If the expression of an identified peptide-associated gene exceeded a certain threshold, it was considered to be a neoantigen. We investigated the association of neoantigen origin proteins, which only occurred in the LN-positive group, with GeneMANIA in the Cytoscape software. For clonality information, the ABSOLUTE algorithm^34^ was used to measure the fraction of cancer cells (CCF) per mutation in the TCIA pipeline. A mutation was classified as clonal if the CCF was >0.95 with probability >0.5, and the mutation was otherwise considered subclonal.

For gene expression data, we downloaded the level-3 RNA-seq FPKM dataset. The number of fragments per kilobase of transcript per million mapped reads is represented. The CIBERSORT^35^ algorithm was used to infer the TIL proportions of the tumor microenvironment. The LM22 dataset was downloaded from the CIBERSORT website (https://cibersort.stanford.edu/download.php), and it consisted of 22 distinct immune cell types and was constructed from the gene expression profiles of these cell types. DESeq2^36^, limma^37^ and TCGAanalyze_DEA function in TCGAbiolinks were used for DEG analysis. In the DESeq2 and TCGAanalyze_DEA methods, the criteria for DEGs were fold-change >2 (or <0.5) and adjusted p-value <0.01. The cutoff in the limma method was fold-change >1.5 (or <0.67) and adjusted *p-value* < 0.05. We kept the intersected genes as the final DEGs. The DESeq2 and TCGAanalyze DEA function in TCGAbiolinks use the count data directly for DEG analysis. While in the limma method, voom function was used to transform count data to log2-counts per million (logCPM) for the linear model. The logCPM was used to transform expression data for heatmap visualization. The DAVID web service^38^ was used for DEG functional annotation and functional clustering. The FGnet^39^ R package was used for functional clustering visualization.

## Electronic supplementary material

Table S1
