# Supplementary material for: Low mutation and neoantigen burden and fewer effector tumor infiltrating lymphocytes correlate with breast cancer metastasization to lymph nodes
Source: Sci Rep. 2019 Jan 22;9:253. doi: 10.1038/s41598-018-36319-x (PMC6342949; doi:10.1038/s41598-018-36319-x)
Supplement: Supplementary file 1 — Table S1 [file 41598_2018_36319_MOESM1_ESM.doc]

**Low mutation and neoantigen burden and fewer effector tumor infiltrating lymphocytes correlate with breast cancer metastasization to lymph nodes**

Zhigang Wang^1^ , Wei Liu^2^ , Chong Chen^3^ , Xiaolin Yang^1^ , Yunping Luo^3^,* , and Bailin

Zhang4,^*^

^1^ Dept. of Biomedical Engineering, Institute of Basic Medical Sciences and School of Basic Medicine, Peking Union Medical College and Chinese Academy of Medical Sciences, Beijing, China

^2^ Dept. of Anatomy and Histology, Institute of Basic Medical Sciences and School of Basic Medicine, Peking Union Medical College and Chinese Academy of Medical Sciences, Beijing, China

^3^ Dept. of Immunology, Institute of Basic Medical Sciences and School of Basic Medicine, Peking Union Medical College and Chinese Academy of Medical Sciences, Beijing, China

^4^ Dept. of Breast Surgery, Cancer Institute/Hospital, Chinese Academy of Medical Sciences and Peking Union Medical College, Beijing, China

^3,*^ ypluo@ibms.pumc.edu.cn

^4,*^ bailin_zhang@cicams.ac.cn

**Table S1**. Tumor infiltrated cell fractions.

| cell_type | Group | cibersort_LM22 | fair_LM22 |
| --- | --- | --- | --- |
| Memory B cell | LN_pos | 0.007502655 | 0.004647788 |
| Naive B cell | LN_pos | 0.022236283 | 0.028416814 |
| Activated dendritic cell | LN_pos | 0.013199115 | 0.024682301 |
| Resting Dendritic cell | LN_pos | 0.031290265 | 0.032915929 |
| Eosinophil | LN_pos | 6.99E-05 | 9.20E-05 |
| Macrophage M0 | LN_pos | 0.124212389 | 0.088990265 |
| Macrophage M1 | LN_pos | 0.111447788 | 0.190479646 |
| Macrophage M2 | LN_pos | 0.149602655 | 0.155390265 |
| Activated mast cells | LN_pos | 0.007529204 | 0.010107965 |
| Resting mast cells | LN_pos | 0.150161062 | 0.088288496 |
| Monocyte | LN_pos | 0.000616814 | 0.005138053 |
| Resting Natural killer cell | LN_pos | 0.018066372 | 0.021383186 |
| Activated Natural killer cell | LN_pos | 0.000730973 | 0.000699115 |
| Neutrophil | LN_pos | 0.010459292 | 0.009815044 |
| Plasma cells | LN_pos | 0.069746903 | 0.046353097 |
| Activated memory CD4 T cell | LN_pos | 0.012122124 | 0.005833628 |
| Resting memory CD4 T cell | LN_pos | 0.054295575 | 0.041350442 |
| Naive CD4 T cell | LN_pos | 0.012613274 | 0.010053982 |
| CD8 T cell | LN_pos | 0.012199115 | 0.011154867 |
| T follicular helper cell | LN_pos | 0.059900885 | 0.079668142 |
| Gamma delta T cell | LN_pos | 0.125752212 | 0.137626549 |
| Regulatory T cell | LN_pos | 0.006238938 | 0.00690531 |
| Memory B cell | LN_neg | 0.011996094 | 0.007532813 |
| Naive B cell | LN_neg | 0.019640625 | 0.02151875 |
| Activated dendritic cell | LN_neg | 0.013685938 | 0.030186719 |
| Resting Dendritic cell | LN_neg | 0.024178125 | 0.026690625 |
| Eosinophil | LN_neg | 4.84E-05 | 0.000115625 |
| Macrophage M0 | LN_neg | 0.174922656 | 0.117416406 |
| Macrophage M1 | LN_neg | 0.110136719 | 0.183951563 |
| Macrophage M2 | LN_neg | 0.156022656 | 0.16700625 |
| Activated mast cells | LN_neg | 0.01181875 | 0.021653125 |
| Resting mast cells | LN_neg | 0.099189844 | 0.05890625 |
| Monocyte | LN_neg | 0.000450781 | 0.003185938 |
| Resting Natural killer cell | LN_neg | 0.019584375 | 0.024467969 |
| Activated Natural killer cell | LN_neg | 0.000305469 | 0.003457031 |
| Neutrophil | LN_neg | 0.009733594 | 0.009650781 |
| Plasma cells | LN_neg | 0.065825781 | 0.045367188 |
| Activated memory CD4 T cell | LN_neg | 0.024278906 | 0.011647656 |
| Resting memory CD4 T cell | LN_neg | 0.044072656 | 0.032389844 |
| Naive CD4 T cell | LN_neg | 0.011684375 | 0.012317188 |
| CD8 T cell | LN_neg | 0.012739063 | 0.01095 |
| T follicular helper cell | LN_neg | 0.068602344 | 0.08578125 |
| Gamma delta T cell | LN_neg | 0.116346875 | 0.121604688 |
| Regulatory T cell | LN_neg | 0.004730469 | 0.004202344 |
| Memory B cell | LN_pos_erminus | 0.01186 | 0.009705 |
| Naive B cell | LN_pos_erminus | 0.02702 | 0.02198 |
| Activated dendritic cell | LN_pos_erminus | 0.02262 | 0.036005 |
| Resting Dendritic cell | LN_pos_erminus | 0.041075 | 0.03795 |
| Eosinophil | LN_pos_erminus | 0.000395 | 0 |
| Macrophage M0 | LN_pos_erminus | 0.14903 | 0.08981 |
| Macrophage M1 | LN_pos_erminus | 0.134435 | 0.22838 |
| Macrophage M2 | LN_pos_erminus | 0.12621 | 0.13598 |
| Activated mast cells | LN_pos_erminus | 0.010045 | 0.01832 |
| Resting mast cells | LN_pos_erminus | 0.07675 | 0.041715 |
| Monocyte | LN_pos_erminus | 0.001095 | 0.00282 |
| Resting Natural killer cell | LN_pos_erminus | 0.0164 | 0.01215 |
| Activated Natural killer cell | LN_pos_erminus | 0 | 0 |
| Neutrophil | LN_pos_erminus | 0.00991 | 0.01088 |
| Plasma cells | LN_pos_erminus | 0.060405 | 0.036985 |
| Activated memory CD4 T cell | LN_pos_erminus | 0.021425 | 0.00892 |
| Resting memory CD4 T cell | LN_pos_erminus | 0.03267 | 0.02499 |
| Naive CD4 T cell | LN_pos_erminus | 0.01338 | 0.00257 |
| CD8 T cell | LN_pos_erminus | 0.01798 | 0.018675 |
| T follicular helper cell | LN_pos_erminus | 0.08 | 0.11102 |
| Gamma delta T cell | LN_pos_erminus | 0.14097 | 0.144715 |
| Regulatory T cell | LN_pos_erminus | 0.006315 | 0.00646 |
| Memory B cell | LN_neg_erminus | 0.0168 | 0.012786957 |
| Naive B cell | LN_neg_erminus | 0.0153 | 0.011228261 |
| Activated dendritic cell | LN_neg_erminus | 0.022141304 | 0.052028261 |
| Resting Dendritic cell | LN_neg_erminus | 0.017441304 | 0.0206 |
| Eosinophil | LN_neg_erminus | 0.000134783 | 1.96E-05 |
| Macrophage M0 | LN_neg_erminus | 0.211030435 | 0.130095652 |
| Macrophage M1 | LN_neg_erminus | 0.117604348 | 0.188173913 |
| Macrophage M2 | LN_neg_erminus | 0.152245652 | 0.161721739 |
| Activated mast cells | LN_neg_erminus | 0.008845652 | 0.02718913 |
| Resting mast cells | LN_neg_erminus | 0.04388913 | 0.024541304 |
| Monocyte | LN_neg_erminus | 0.000856522 | 0.001095652 |
| Resting Natural killer cell | LN_neg_erminus | 0.022352174 | 0.019726087 |
| Activated Natural killer cell | LN_neg_erminus | 7.39E-05 | 0.004180435 |
| Neutrophil | LN_neg_erminus | 0.007697826 | 0.011584783 |
| Plasma cells | LN_neg_erminus | 0.070730435 | 0.046908696 |
| Activated memory CD4 T cell | LN_neg_erminus | 0.039847826 | 0.019076087 |
| Resting memory CD4 T cell | LN_neg_erminus | 0.029493478 | 0.025863043 |
| Naive CD4 T cell | LN_neg_erminus | 0.006902174 | 0.003254348 |
| CD8 T cell | LN_neg_erminus | 0.017958696 | 0.015026087 |
| T follicular helper cell | LN_neg_erminus | 0.079836957 | 0.100343478 |
| Gamma delta T cell | LN_neg_erminus | 0.114241304 | 0.120767391 |
| Regulatory T cell | LN_neg_erminus | 0.004580435 | 0.003784783 |
| Memory B cell | LN_pos_erplus | 0.006414458 | 0.003608434 |
| Naive B cell | LN_pos_erplus | 0.021918072 | 0.03053494 |
| Activated dendritic cell | LN_pos_erplus | 0.010512048 | 0.020707229 |
| Resting Dendritic cell | LN_pos_erplus | 0.026603614 | 0.030243373 |
| Eosinophil | LN_pos_erplus | 0 | 0.000125301 |
| Macrophage M0 | LN_pos_erplus | 0.123949398 | 0.091838554 |
| Macrophage M1 | LN_pos_erplus | 0.106901205 | 0.182640964 |
| Macrophage M2 | LN_pos_erplus | 0.150324096 | 0.156225301 |
| Activated mast cells | LN_pos_erplus | 0.00776506 | 0.008585542 |
| Resting mast cells | LN_pos_erplus | 0.166071084 | 0.097024096 |
| Monocyte | LN_pos_erplus | 0.000498795 | 0.005285542 |
| Resting Natural killer cell | LN_pos_erplus | 0.017791566 | 0.02273012 |
| Activated Natural killer cell | LN_pos_erplus | 0.000995181 | 0.000937349 |
| Neutrophil | LN_pos_erplus | 0.0103 | 0.009575904 |
| Plasma cells | LN_pos_erplus | 0.069377108 | 0.046816867 |
| Activated memory CD4 T cell | LN_pos_erplus | 0.009827711 | 0.00520241 |
| Resting memory CD4 T cell | LN_pos_erplus | 0.059942169 | 0.045320482 |
| Naive CD4 T cell | LN_pos_erplus | 0.012551807 | 0.012392771 |
| CD8 T cell | LN_pos_erplus | 0.010393976 | 0.010042169 |
| T follicular helper cell | LN_pos_erplus | 0.055989157 | 0.072785542 |
| Gamma delta T cell | LN_pos_erplus | 0.125198795 | 0.140216867 |
| Regulatory T cell | LN_pos_erplus | 0.006662651 | 0.007143373 |
| Memory B cell | LN_neg_erplus | 0.00812027 | 0.003975676 |
| Naive B cell | LN_neg_erplus | 0.023286486 | 0.027625676 |
| Activated dendritic cell | LN_neg_erplus | 0.008095946 | 0.016586486 |
| Resting Dendritic cell | LN_neg_erplus | 0.026867568 | 0.029967568 |
| Eosinophil | LN_neg_erplus | 0 | 0.000187838 |
| Macrophage M0 | LN_neg_erplus | 0.151581081 | 0.108895946 |
| Macrophage M1 | LN_neg_erplus | 0.109235135 | 0.186082432 |
| Macrophage M2 | LN_neg_erplus | 0.153402703 | 0.166843243 |
| Activated mast cells | LN_neg_erplus | 0.013009459 | 0.016305405 |
| Resting mast cells | LN_neg_erplus | 0.13257973 | 0.078993243 |
| Monocyte | LN_neg_erplus | 0.000247297 | 0.004109459 |
| Resting Natural killer cell | LN_neg_erplus | 0.019344595 | 0.028336486 |
| Activated Natural killer cell | LN_neg_erplus | 0.000202703 | 0.002112162 |
| Neutrophil | LN_neg_erplus | 0.010764865 | 0.008174324 |
| Plasma cells | LN_neg_erplus | 0.059795946 | 0.043635135 |
| Activated memory CD4 T cell | LN_neg_erplus | 0.016275676 | 0.007874324 |
| Resting memory CD4 T cell | LN_neg_erplus | 0.052540541 | 0.035124324 |
| Naive CD4 T cell | LN_neg_erplus | 0.015783784 | 0.017135135 |
| CD8 T cell | LN_neg_erplus | 0.010871622 | 0.0096 |
| T follicular helper cell | LN_neg_erplus | 0.063639189 | 0.079885135 |
| Gamma delta T cell | LN_neg_erplus | 0.120571622 | 0.124836486 |
| Regulatory T cell | LN_neg_erplus | 0.003775676 | 0.003718919 |
